# The Notification of Tuberculosis

**Published:** 1896-07-04

**Authors:** 


### The Hospital

AN INSTITUTIONAL JOURNAL OF

The Medical Sciences and Hospital Administration,

WITH

AN EXTRA NURSING SUPPLEMENT.

VOL. XX.—No. 510.] [JULY 4, 1896.

### THE NOTIFICATION OF TUBERCULOSIS.

FROM measles to tuberculosis is but a small step in the eyes of some. Both are zymotic diseases, both are very widely spread, both of them cause a large loss of life which may fairly be considered to be avoidable, while both are outside the provisions of the Notification Act as usually interpreted, and both are allowed to run their course without any attempt on the part of the sanitary authorities to isolate those who are affected by them.

Nay, they are alike in this also, that in regard to both of them one great argument against enforcing notification is that it would be quite impossible to provide for that complete hospital isolation which is held by many to be the natural and logical conclusion to which notification is but a step.

It is not, therefore, to be wondered at that the article which appeared in THE HOSPITAL on June 6th, on "The Municipal Control of Measles," should have attracted the attention of those sanitarians who look upon tuberculosis as the disease of all others that most deserves municipal control; for so far as we were able to show that, in regard to measles, notification had other uses besides paving the way to hospital isolation, so it was easy to assume that in regard to tuberculosis also the same thing must hold good, and that, however hopeless the provision of hospital accommodation for all the cases of phthisis must be, notification of it none the less would be desirable. It may be worth while then, having pointed out similarities, to indicate certain differences between these diseases which lead us to think that notification of tuberculosis may rightly be, for the time, put off.

Measles is eminently infectious even amid good surroundings, and this infection may be carried by third parties from the giver to the receiver. Tuberculosis is eminently non-infectious, taking infectivity in the sense in which the word is used in regard to measles, and where it is infectious, this property depends on the surroundings rather than upon the patient himself, and these bad surroundings it is the duty of the sanitary authorities to know about, quite irrespective of their eventuating in the production of phthisis.

Measles is an affair of short duration, and the origin of the disease can be searched for with a reasonable chance of success; while tuberculosis is a disease of extremely long duration, the initial attack occurring sometimes months, and even years, before any advice is sought, or, if advice be sought, before it could be definitely asserted that tuberculosis is present.

Measles is an acute disease, during the progress of which the patient is helpless and requires nursing and assistance, while tuberculosis is a disease during the greater portion of the course of which patients are in many cases active members of society, earning their living and supporting their families, and both willing and able strongly to resent any interference with their liberty.

Measles, again, being an acute disease each case falls into the hands of one doctor, on whom the responsibility of notifying can easily be fixed; whereas tuberculosis is a slow affair and the patient goes to twenty doctors in the course of its progress, and, unless we are prepared to admit multiple notifications, no one could be made responsible and the whole thing would become a dead letter.

But the great argument against at present undertaking notification of tuberculosis lies in the fact that, while its difficulties are great and its advantages problematical, there are so many other things to do for its suppression, the difficulties of which are by no means insurmountable and the advantages of which are by no means problematical. The dirt, the overcrowding, the darkness, and the want of air in the dwellings of the people, the surroundings under the influence of which alone the infectivity of tuberculosis becomes a serious matter, are as capable of reformation without notification as with it; and if they are incapable of reformation no amount of notification can be expected to do much good. Then a careful inspection of the meat supply, the abolition of private slaughter-houses and the official inspection of every slaughtered animal, is an affair quite independent of notification. The same also in regard to the inspection of milk-supplying cows. It would seem ridiculous to labour at notification and invent a tyrannical system of isolation for doubtfully infective patients, and all the while allow our farmers to supply us under the guise of milk with a solution of tuberculous infection.

It must also be remembered that it is very premature to urge the general notification of tuberculous diseases when we have not yet arrived at the point of providing a refuge for those who are known and acknowledged to be consumptive, who have got to that sad point when work is no longer possible to them, when they can but totter along the sunny side of our streets, spitting as they go, or have to content themselves with spending the weary remainder of their lives sitting by the cottage fire or lying in the crowded cottage bedroom, or in the still more crowded London tenement—spitting, and infecting others as they sink themselves. These are the people who are infectious, and it is, no doubt, by the presence of these invalids, who by the charity of their relatives are thus kept out of the workhouse, that the dwellings of the poor too often become the nurseries of consumption.

These people are perfectly well known, and they would willingly go to hospitals where they might live out their lives in comfort, and save their relatives from danger; but there is no such refuge yet open to them, and we repeat that until some such refuge is provided, until some more active steps are taken to improve the lower levels of the dwellings of the poor, and so long as we are content to eat the uninspected food we do eat, and to drink the milk from uninspected cows which is now provided for us, it is premature to talk of the notification of tuberculosis.

